# Impact of the representation of stomatal conductance on model projections of heatwave intensity

**DOI:** 10.1038/srep23418

**Published:** 2016-03-21

**Authors:** Jatin Kala, Martin G. De Kauwe, Andy J. Pitman, Belinda E. Medlyn, Ying-Ping Wang, Ruth Lorenz, Sarah E. Perkins-Kirkpatrick

**Affiliations:** 1 School of Veterinary and Life Sciences - Environmental and Conservation Sciences, Murdoch University, Perth, Western Australia, Australia; 2 Macquarie University, Department of Biological Sciences, Sydney, Australia; 3 Australian Research Council Centre of Excellence for Climate Systems Science and Climate Change Research Center, University Of New South Wales, New South Wales, Australia; 4 Hawkesbury Institute for the Environment, University of Western Sydney, Sydney, Australia; 5 Commonwealth Scientific and Industrial Research Organisation, Ocean and Atmosphere Flagship, Aspendale, Victoria, Australia

## Abstract

Stomatal conductance links plant water use and carbon uptake, and is a critical process for the land surface component of climate models. However, stomatal conductance schemes commonly assume that all vegetation with the same photosynthetic pathway use identical plant water use strategies whereas observations indicate otherwise. Here, we implement a new stomatal scheme derived from optimal stomatal theory and constrained by a recent global synthesis of stomatal conductance measurements from 314 species, across 56 field sites. Using this new stomatal scheme, within a global climate model, subtantially increases the intensity of future heatwaves across Northern Eurasia. This indicates that our climate model has previously been under-predicting heatwave intensity. Our results have widespread implications for other climate models, many of which do not account for differences in stomatal water-use across different plant functional types, and hence, are also likely under projecting heatwave intensity in the future.

Heatwaves are extreme phenomena that have major impacts on environmental, social, health and economic systems^1^. We define heatwaves as a series of three or more consecutive days during which daily maximum temperatures are higher than the calendar-day 90^th^ percentile^2^. The frequency, intensity and duration of heatwaves are increasing in many parts of the globe^3^,^4^,^5^. Observations have highlighted an increase in the length of European heatwaves^6^ and the frequency of heatwave occurrence in China^7^ and Australia^2^,^8^. For example, the 2003 summer heatwave affected much of Western Europe and likely provided a precursor to future extremes across this region^9^. Many of these observed large-scale heatwaves have been linked to human activity via global warming^10^,^11^.

Future warming linked with increases in greenhouse gases is expected to increase the frequency, intensity and duration of heatwaves further^5^,^12^, particularly across the mid-latitudes including North America and Europe^13^,^14^. Heatwaves are associated with large-scale synoptic states^15^,^16^, which are influenced by modes of climate variability^17^. However, it is now well established from observational^18^ and modelling studies^19^,^20^ that heatwaves are also strongly modulated by the land surface if the synoptic scale weather generates persistent anticyclonic patterns and the planetary boundary-layer strongly couples the land to the atmosphere over consecutive days^21^. Under these circumstances, heatwaves intensify as desiccated soils and a surface radiation balance dominated by the exchange of sensible heat is coupled with the boundary-layer to lead to events such as the “mega-heatwaves” experienced in Europe during 2003 and 2010^19^,^21^. Although the detailed role of the land surface on the exchange of water and energy during heatwaves remains uncertain^22^, there is evidence that capturing the detailed connection between the land and the atmosphere, and how soil moisture impacts the surface energy balance to moderate or intensify heat waves, is necessary to produce realistic simulations of these phenomena^19^.

Within climate models, land surface models (LSMs) simulate soil moisture and partition available radiation at the surface between sensible and latent heat fluxes^23^. For vegetated surfaces, in particular over forests, the latent heat flux is principally controlled by stomata, as plants exchange water for carbon. Our ability to accurately simulate how soil moisture states and soil moisture variability affects heatwaves, therefore relies at least in part, on accurately modelling stomatal conductance (g_s_) under current and future CO_2_ concentrations. At the leaf scale, experiments commonly find that increasing CO_2_ results in increased photosynthesis^24^,^25^ and reduced water loss via lower g_s_^26^,^27^,^28^. However, there is increasing evidence^29^,^30^ that we cannot easily transfer our leaf/canopy level understanding of the response of transpiration due to CO_2_, to ecosystem scales. Nevertheless, any CO_2_-induced change in transpiration and/or soil “water-savings”, has the potential to alter future soil moisture state, soil moisture variability, and transpiration, which may then feedback on the development of heatwaves over several days^21^. Given that heatwaves are associated with synoptic state and persistent anticyclonic conditions or so-called “blocking/persistent highs”^31^,^32^, these feedbacks are more likely to affect heatwave intensity than duration or frequency.

To date, the representation of g_s_ in LSMs has been largely based on empirical models^33^,^34^,^35^. These models typically assume that differences in plant water use strategy are only tied to the photosynthetic pathway (C3 vs. C4). This assumption is not supported by experimental evidence; instead leaf level measurements suggest that plant water use strategies vary among species (or plant functional types, PFTs)^36^. Ignoring these differences among PFTs will likely result in errors in the simulated flux of moisture to the atmosphere. A recent collation of a global database of leaf-level g_s_ measurements^36^ from 319 species across 56 field studies was used to parameterise differences in plant water use strategy among PFTs within the Community Atmosphere Biosphere Land Exchange (CABLE) model^37^. Parameters were estimated for each of the models PFTs by fitting Eq. 1 (see Methods) to this leaf-level dataset using a non-linear mixed effects model^38^.

This new g_s_ model^27^,^36^ is similar in functional form to the previous empirical model^35^ used in CABLE and many other LSMs but is derived following optimal stomal theory. Consequently, model parameters carry biological meaning and can be hypothesised to vary with climate and plant water use strategy^27^. Such variations are supported by experimental data^36^. Offline CABLE simulations^38^ and coupled land-atmosphere simulations^39^ performed using the Australian Community Climate and Earth Systems Simulator (ACCESS1.3b)^40^, showed that this parameterisation led to a reduction in transpiration (up to 1 mm day^−1^) across boreal regions, which resulted in an increase in daily minimum and maximum temperatures (by up to 1 °C). These changes in contemporary simulations of water fluxes and daily warm temperature extremes were an improvement in the model’s climatology in comparison to observations during the boreal summer, especially over Eurasia^39^.

We extend our previous work^38^,^39^ to examine how an alterantive g_s_ model, constrained by a global synthesis of leaf-level measurements, impacts upon future simulations of the likely incidence of heatwaves. We use the “business as usual” emission scenario (Representative Concentration Pathway 8.5 (RCP8.5))^41^ with the ACCESSv1.3 climate model. To the best of our knowledge, this is the first paper to implement a g_s_ model within a global climate model focussing on future climate simulations, where the g_s_ model parameters vary per PFT and are derived from best available data. We focus specifically on Eurasia for several reasons. Firstly, this is the region where the new g_s_ scheme improved ACCESS’s climatology of evaporation and warm extremes^39^. Secondly, a previous evaluation of ACCESS’s simulations of extremes has shown large biases in extreme temperatures linked to clouds over North America^42^ and hence we avoid analysing this continent. Thirdly, Eurasia was shown to be sensitive to the parameterization of g_s_ in earlier ACCESS experiments^39^ and finally, work by many researchers^19^,^21^,^43^ hints at this region being susceptible to large changes in warm extremes and heatwaves in the future.

## Results

**Figure 1:**
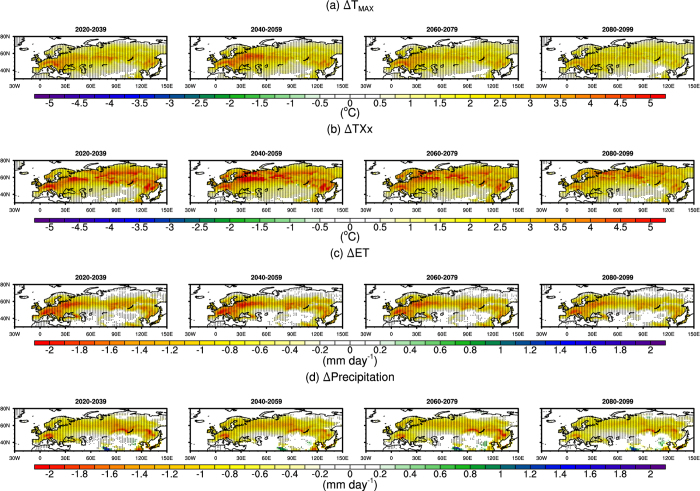
Difference (Experiment minus Control) in mean Boreal summer (June-July-August) (**a**) daily maximum temperature (T_MAX_, top row), (**b**) warmest maximum temperature (TXx, middle row), and (**c**) evapotranspiration (ET, bottom row), and (**d**) precipitation (mm day^−1^), averaged over 20 year intervals between 2020–2099. Stippling shows regions where differences are statistically significant at the 95% level using the student’s t-test and the false discovery method for field significance. This figure was created using NCLV6.2.1 (http://www.ncl.ucar.edu/).

We first examine changes in warm extremes and surface moisture fluxes as illustrated in Fig. 1 showing the difference in mean Boreal summer (June-July-August) daily maximum temperature (T_MAX_, Fig. 1a), warmest yearly maximum temperature (TXx, Fig. 1b), and evapotranspiration (ET, Fig. 1c), averaged over 20 year intervals (2020–2099), between the new and the default g_s_ scheme (i.e., Experiment minus Control). T_MAX_ increases commonly by ∼1 °C but by more than 1.5 °C over Western Europe and 2 °C in some regions. The impact of the new g_s_ scheme on TXx is larger, reaching 5 °C over widespread regions. Not surprisingly, there is a strong similarity between the patterns of temperature increases, decreases in ET (Fig. 1c), and subsequent decrease in precipitation (Fig. 1d), consistent with our previous work^39^. We note that the difference in both T_MAX_ and TXx between models is largest during the period 2040–2059 and decreases towards the end of the century. One possible explanation for this decrease is that at high leaf temperatures (ca. 30 °C), photosynthesis and stomatal conductance (and thus transpiration) are reduced due to photosynthetic inhibition (Fig. S2). This response to high temperature minimises the differences in transpiration between the models that originally resulted from the more conservative water use paramaterisation in the new scheme.

Furthermore, the two g_s_ schemes have different sensitivities to vapor pressure deficit (VPD), with the default model showing stronger sensitivity at high VPD (>3 kPa)^38^. Thus, as dryland expansion accelerates under climate change^44^, and the air temperature and VPD increase towards the end of the 21^st^ century, the difference in predicted transpiration between the two models becomes smaller (Fig. S2 and related text), which potentially accounts for the smaller effect on T_MAX_ and TXx compared to earlier in the century. Nevertheless, there are still large differences between the models across most of Eurasia at the end of the century (2 to 4 °C for TXx).

**Figure 2:**
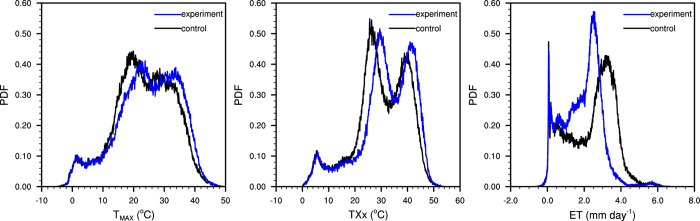
Probability distribution function (PDF, %) of monthly mean Boreal summer (June-July-August) daily maximum temperature (T_MAX_, left plot) warmest maximum temperature (TXx, middle plot), and evapotranspiration (ET, right plot) over the period 2020–2099. Results using the new g_s_ are shown in blue (i.e., experiment), and the default g_s_ scheme is in black (i.e., the control). This figure was created using NCLV6.2.1 (http://www.ncl.ucar.edu/).

The increases in T_MAX_ and TXx and a decrease in ET can be clearly seen in the probability density functions (PDFs, Fig. 2). There is a clear shift to the right for the PDF of T_MAX_ and TXx, but the limits of the lower and upper tails are mostly unchanged. The new g_s_ scheme does not lead to the emergence of temperatures not previously experienced across the region; rather, it leads to a much more frequent occurrence of hot temperatures. Clearly, this change is linked to a shift in the PDF of ET to the left, such that ET exceeding 4 mm day^−1^ is rare with the new g_s_ scheme, but common using the old scheme.

**Figure 3:**
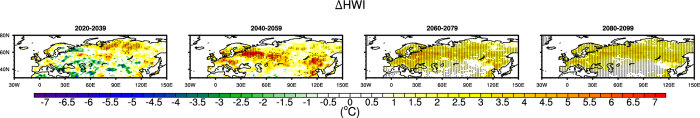
Same as in Fig. 1 except showing the change in heatwave intensity (HWI). This figure was created using NCLV6.2.1 (http://www.ncl.ucar.edu/).

We next examined the influence of the change in g_s_ on heatwave duration, frequency and intensity (see Methods for definition). The changes in heatwave duration and frequency were very small, but changes in heatwave intensity (HWI) were large (Fig. 3). During the earlier part of the century (2020–2039), there are regions of both increases and reductions in HWI indicating that the forcing associated with the change in g_s_ is commonly smaller than internal model variability. However, by 2040–2059, the new scheme results in an increase in HWI everywhere, with particularly large increases over western Europe, western Russia and eastern China, where HWI increases by 6–7 °C. Similarly to the changes in T_MAX_ and TXx, the magnitude of the increase in HWI decreases towards the end of the century, but remains higher than 5 °C in many regions.

### Discussion and Conclusion

The increase in future (2020–2099) simulated TXx resulting from changing the representation of g_s_ is approximately 4–5 °C over Western Europe. This sensitivity to g_s_ can be put into context by recognising that this change is equivalent to more than half the increase projected under RCP8.5^41^ (>1370 ppm CO_2_ equivalent in 2100) by an ensemble of climate models for 2081–2100^45^. The change is similar to estimates reported for RCP4.5^41^ (~650 ppm CO_2_ equivalent at stabilization after 2100) and higher than those reported for RCP2.6^41^ (~490 equivalent before 2100 and declining) by 2081–2100^46^. It is also similar in magnitude to the estimate reported for the change in heatwave intensity under RCP8.5^47^. The increases in TXx due to the change in the g_s_ model and parameterisation are therefore of the size reported for large increases in greenhouse gases.

Over western and northern Europe, the changes in TXx and heatwave intensity due to a change in the representation of g_s_ as reported here are similar in terms of both pattern and intensity when compared to studies which have linked these changes to projected increases in greenhouse gases^46^,^47^. There are regions where the improved parameterization of g_s_ led to increases in temperature and improved simulations^39^, particularly between around 45–60°N. The increases predominately occurred across regions defined as evergreen needleleaf forest, Tundra, and crop PFTs.

The stomatal parameterisation we used in ACCESS accounts for differences in stomatal behaviour between PFTs and is supported by a global synthesis of leaf-level stomatal data^36^, in line with both predictions from optimal stomatal theory^27^,^48^ and the leaf and wood economic spectrum^49^,^50^. This empirical basis lends support to the robustness of these model simulations, which highlight the role of stomatal conductance in influencing future heatwaves. Nevertheless, some uncertainties remain. First, the data behind this parameterisation are measured at leaf scale; it has not been confirmed that the differences among PFTs observed at this scale also emerge at canopy/ecosystem scale. In light of our results, there is an urgent need for future work which tests how the stomatal parameterisation (g_1_, the sensitivity of the conductance to the assimilation rate, see material and methods) scales from the leaf to the canopy/ecosystem. Secondly, we have assumed all vegetation to have the same drought sensitivity. Observations suggest that vegetation adapted to different hydroclimates have different sensitivity^51^, which has significant consequences for ecosystem-scale water flux during drought periods^52^. A generic parameterisation for varying drought sensitivity across different vegetation types is another important priority.

We note a further significant caveat to our study: the ACCESS 1.3b climate model, in common with all climate models, has biases in its simulation of extremes^42^. The new g_s_ parameterisation resolves some of these biases, at both site^38^ and global scales^38^,^39^. We also note that heatwaves are coupled phenomenon linking large-scale synoptic conditions, persistence, boundary layer coupling and land processes^21^. While ACCESS 1.3b is similar to other models in its representation of land-atmosphere coupling strength^53^ it remains a limitation to our study that we used a single climate model. We therefore encourage other groups to repeat our experiments to see if they can be generalized. Our results are also influenced by our use of a prescribed monthly climatology of leaf area index (LAI) derived from remote sensing estimates (see Methods). By prescribing the LAI, we are not allowing increases in leaf area due to CO_2_ to reduce any CO_2_ induced “water savings”. A model inter-comparison study^54^ which examined the response to elevated CO_2_ at two free-Air CO_2_ enrichment experiments found that even when LAI was not prescribed, the land surface component of ACCESS, i.e., CABLE, predicted modest changes in LAI (~5% increase). This result suggests that the use of prescribed LAI is unlikely to affect the results shown here for ACCESS, but clearly this may vary in other climate models. As both simulations prescribed the same LAI, the result is robust to assumptions of leaf area and CO_2_, and instead highlights the direct impact of the change in g_s_ scheme and parameterization. Nevertheless, we plan to investigate the influence of prognostic LAI between the two schemes in future work.

The impact of the revised g_s_ scheme on heatwave intensity is confronting, with increases of 5 °C (2040–2059). These increases are additive to those likely caused by increasing greenhouse gases over the same period^47^. The magnitude of these changes is large when compared to studies which have investigated the influence of soil moisture and vegetation dynamics on heatwaves. For example, lowering soil moisture by 25% for the 2003 European heatwaves is reported to lead to a maximum increase of 2 °C^20^, and other studies report changes of +0.5 °C by increasing LAI^55^ and ±1.5 °C due to dynamic phenology^56^. Our results are inevitably model-specific, but if confirmed by other groups, the current systematic under-estimation of future increases in heatwave intensity will have significant implications for socio-economic and environmental systems. We note that our revised parameterization of g_s_ had no impact on the frequency or duration of heatwaves, since these are primarily driven by larger-scale synoptic-scale processes such as blocking highs^57^ and changing patterns of circulation^58^. However, our results do show that g_s_ strongly affects the intensity of heatwaves over Eurasia and is therefore further evidence that land-atmosphere interactions are an important driver of extreme temperature events.

## Methods

### New representation of stomatal conductance

The default g_s_ model^35^ used in ACCESSv1.3b^40^ has been described in detail in the literature^37^. The new g_s_ scheme^27^ follows the form:


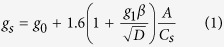


where A is the net assimilation rate (μmol m^−2^ s^−1^), C_s_ (μmol mol^−1^) and D (kPa) are the CO_2_ concentration and the vapour pressure deficit at the leaf surface, respectively, and g_0_ (mol m^−2^ s^−1^), and g_1_ (kPa^0.5^) are fitted constants representing the residual stomatal conductance as A rate reaches zero, and the slope of the sensitivity of g_s_ to A, respectively. g_0_ is zero, leaving one key model parameter, g_1_, which theoretically represents the marginal carbon cost of water^27^.

The model was parameterised for the different PFTs (Fig. S1) using a global synthesis of stomatal measurements compiled from 314 species, across 56 field sites, covering the Arctic tundra, boreal regions, temperate forests and tropical rainforest biomes^36^. Values are shown in Table S1. The default g_s_ scheme in CABLE has two fitted parameters which only vary by photosynthetic pathway (C3 versus C4) but not by PFT. More details on the differences between the default and new scheme and the implementation of the new scheme in CABLE can be found in our earlier work^38^,^39^.

### Simulations

The ACCESS model setup is identical to our previous work in evaluating the new g_s_ model under current climate^39^, except that simulations use sea surface temperatures from a previous fully-coupled simulation with ACCESS1.3 driven by the RCP8.5 emission scenario^41^ (official CMIP5 submission). Five ensembles were run; each initialised a year apart, with the default g_s_ scheme (i.e., the control), and the new scheme (i.e., the experiment). All results shown are for the ensemble mean. We performed statistical significance testing of the differences between the experiment and the control using the student’s- t-test at 95% confidence interval, and tested for field significance using the false discovery rate method^59^. Similar to our previous work^39^, nutrient-limited carbon pool dynamics and dynamic phenology were not activated, as the focus was on biophysical effects of the new g_s_ scheme. Leaf area index (LAI) was prescribed as a monthly climatology derived from MODIS estimates. Results are also only shown between 30°W-150°E longitude and 30°N-80°N latitude, corresponding to the region where the new g_s_ scheme improved ACCESS’s climatology of ET and warm extremes when compared to observations^39^.

### Heatwave definition

Following the literature on heatwaves (HWs)^2^, we use thresholds based on percentiles rather than absolute values, with an event defined as temperatures exceeding the 90% percentile of daily maximum temperatures for at least 3 consecutive days. The percentiles are computed for each calendar day over a moving window over a user-defined base period, which is a commonly adopted approach^5^,^43^,^46^,^60^. To account for seasonality, we use a 15-day moving window over a 30-year base period during the first 30 years of the simulation (2020–2049) to provide the baseline. We note that most studies use 1961–1990^46^,^60^; however, although we do have data over this period, these were generated using observed prescribed sea surface temperatures^39^. So, for consistency, we use the first 30 years of our simulation as baseline. The percentiles are computed for each of the 5 ensembles of the control and experiment separately. The HW-duration is the mean length (in days) of heatwave events during summer; the HW-frequency is the number of HW events; the HW-intensity is the mean temperature during the HW events; and the HW-max-intensity is the maximum temperature during the HW events. Indices are averaged across the 5 ensembles for the control and experiment and the ensemble mean difference is shown between the experiment and the control.

## Additional Information

**How to cite this article**: Kala, J. *et al*. Impact of the representation of stomatal conductance on model projections of heatwave intensity. *Sci. Rep.* **6**, 23418; doi: 10.1038/srep23418 (2016).

## Supplementary Material

Supplementary Information
