# Supplementary material for: Impact of the representation of stomatal conductance on model projections of heatwave intensity
Source: Sci Rep. 2016 Mar 21;6:23418. doi: 10.1038/srep23418 (PMC4800495; doi:10.1038/srep23418)
Supplement: Supplementary Information [file srep23418-s1.doc]

# Supplemantary material

# Impact of the representation of stomatal conductance on model projections of heatwave intensity

Jatin Kala^1^, Martin G. De Kauwe^2^, Andy J. Pitman^3^, Belinda E. Medlyn^4^, Ying-Ping Wang^5^, Ruth Lorenz^3^ , and Sarah E. Perkins-Kirkpatrick^3^

1. School of Veterinary and Life Sciences - Environmental and Conservation Sciences, Murdoch University, Perth, Western Australia, Australia and Australian Research Council Centre of Excellence for Climate Systems Science

2. Macquarie University, Department of Biological Sciences, Sydney, Australia

3. Australian Research Council Centre of Excellence for Climate Systems Science and Climate Change Research Center, University Of New South Wales, New South Wales, Australia

4. Hawkesbury Institute for the Environment, University of Western Sydney, Sydney, Australia,

5. Commonwealth Scientific and Industrial Research Organisation, Ocean and Atmosphere Flagship, Aspendale, Victoria, Australia

Corresponding Author: Jatin Kala (J.Kala@murdoch.edu.au)

In the main text we noted that towards the end of the 21 century, simulated fluxes from ACCESS with the the two stomatal conductance schemes became more similar, whereas earlier in the century, there were large differences. One possible explanation for this is shown in Fig. S2 using a simplified model to represent the behaviour of the ACCESS model with the two different stomatal conductance schemes. At high leaf temperatures (ca. 30°C), photosynthesis and stomatal conductance (and thus transpiration) are reduced due to photosynthetic inhibition. This response to high temperature minimises the differences in transpiration between the models that are caused by the parameterisation. Furthermore, the two stomatal schemes have different sensitivities to vapour pressure deficit, with the default model showing stronger sensitivity at high vapour pressure deficits (>3 kPa) (see 41). We also note that for the default scheme in these simplified simulations, transpiration increases as photosynthesis and stomatal conductance tend to zero. This effect occurs because g_0_ is assumed to be positive. However, this effect is over-estimated in the simple model compared to ACCESS because the simple model does not consider any limitations due to available soil water.


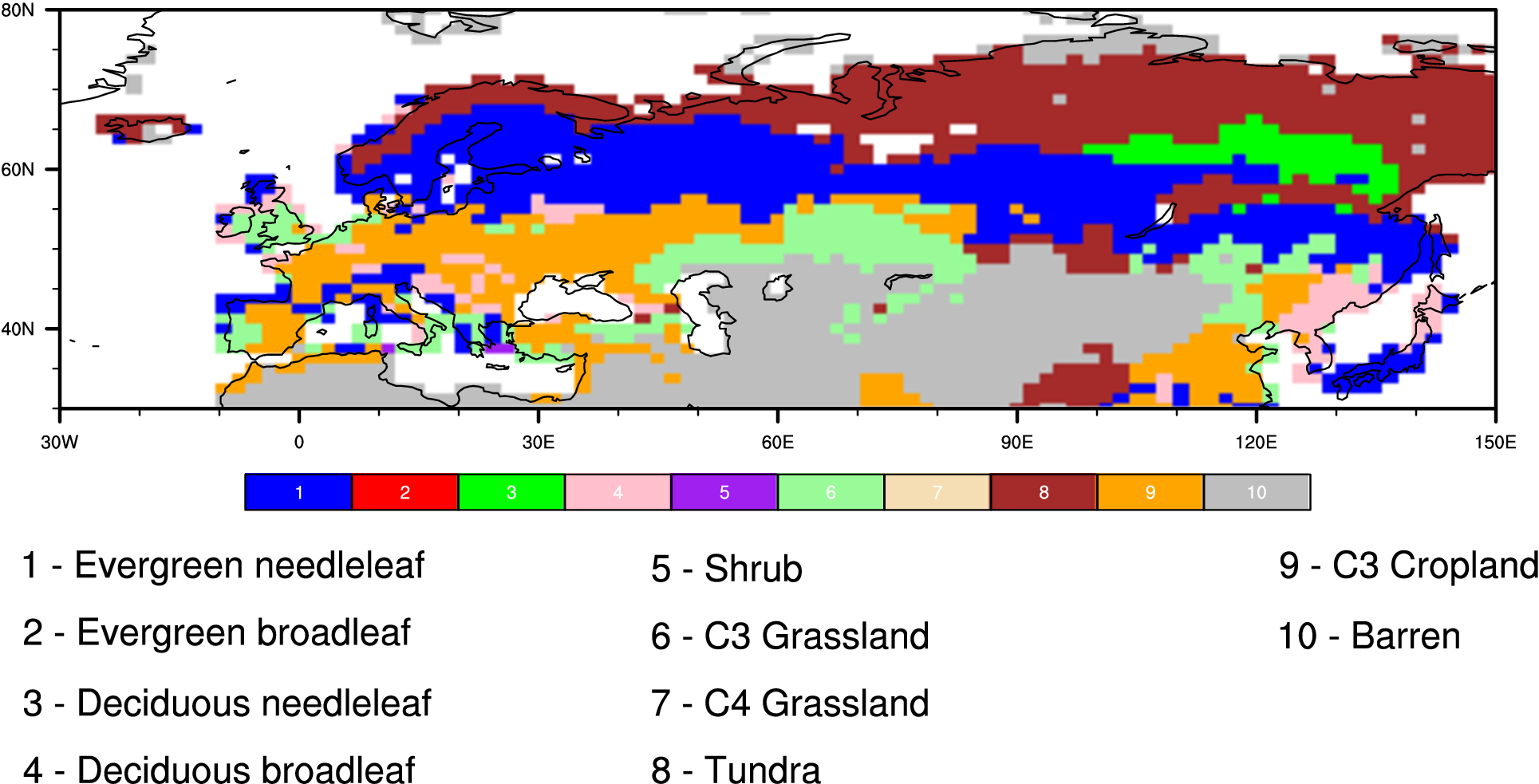


Figure S1: Distribution of dominant plant functional types (PFTs). This figure was created using NCLV6.2.1 (http://www.ncl.ucar.edu/).


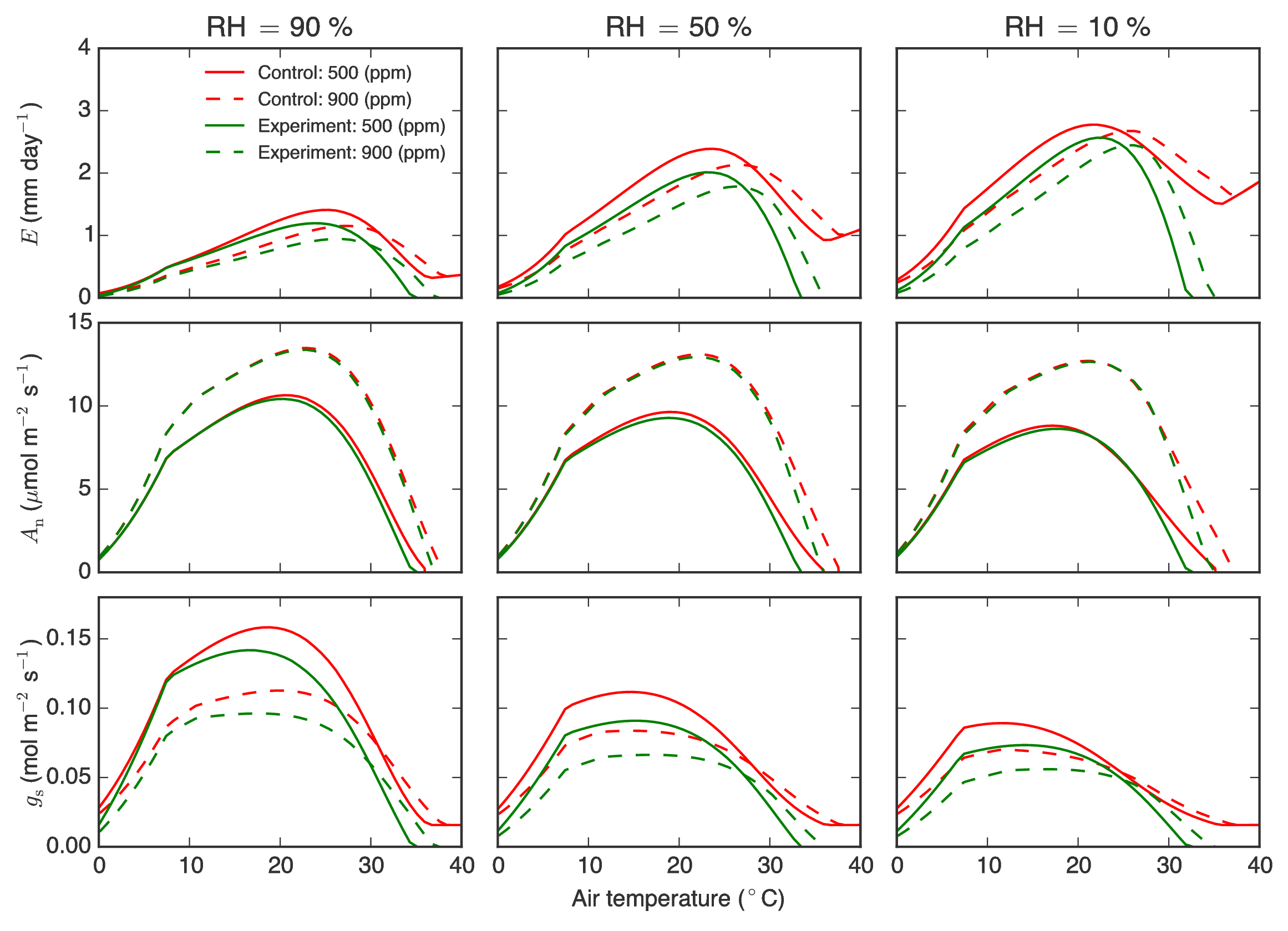
Figure S2: Sensitivity of transpiration (*E*), net leaf assimilation (A_n_) and stomatal conductance (g_s_) to increasing air temperature and decreasing relative humidity at 500 and 900 ppm CO_2_. Simulations are from a model which simultaneously solves leaf level A_n_, *E*, g_s_, and the leaf energy balance (38). These simulations depict idealised leaf-level simulations for the evergreen needleleaf forest plant functional type (Fig. S1), for the default (Control) and new (Experiment) g_s_ scheme. For the default scheme, the residual stomatal conductance as the net assimilation rate reaches zero, g_0_ = 0.01 mol m^-2^ s^-1^; the sensitivity of the conductance to the assimilation rate, g_1_ = 9.0 (-); and the sensitivity of stomatal conductance to vapour pressure deficit, D_0_ = 1.5 kPa. For the new scheme, g_0_ = 0.0 mol m^-2^ s^-1^ and g_1_ = 2.35 kPa^0.5^. In all simulations the maximum carboxylation rate and potential rate of electron transport at 25°C were set to 40.0 and 80.0 μmol m^-2^ s^-1^, respectively. More details on both schemes can be found in our previous work (41) and the idealised model is available at: https://github.com/mdekauwe/coupled_A_gs_leaf_temp_transp

Table S1: Fitted g_1_ values for the PFTs shown in Fig. S1.

| PFT | g_1_ (kPa^0.5^) |
| --- | --- |
| Evergreen needleleaf | 2.35 |
| Evergreen broadleaf | 4.12 |
| Deciduous needleleaf | 2.35 |
| Deciduous broadleaf | 4.45 |
| Shrub | 4.70 |
| C3 grassland | 5.25 |
| C4 grassland | 1.62 |
| Tundra | 2.22 |
| C3 cropland | 5.79 |
